# Pediatric Echocardiographic Nomograms: Twenty Years of Advances—Do We Now Have a Complete and Reliable Tool, or Are Gaps Still Present? An Up-to-Date Review

**DOI:** 10.3390/jcm14155215

**Published:** 2025-07-23

**Authors:** Massimiliano Cantinotti, Pietro Marchese, Guglielmo Capponi, Eliana Franchi, Giuseppe Santoro, Alessandra Pizzuto, Nadia Assanta, Raffaele Giordano

**Affiliations:** 1 Fondazione CNR-Regione Toscana G. Monasterio, 54100 Massa, Italy; cantinotti@ftgm.it (M.C.); pitrino91@gmail.com (P.M.); gcapponi@ftgm.it (G.C.); eliana.franchi@ftgm.it (E.F.); giuseppe.santoro@ftgm.it (G.S.); apizzuto@ftgm.it (A.P.); assanta@ftgm.it (N.A.); 2 Advanced Biomedical Sciences, Cardiac Surgery, University of Naples Federico II, Via Pansini, 5, 80131 Naples, Italy

**Keywords:** echocardiography, children, nomograms

## Abstract

Echocardiography is the primary imaging modality for diagnosing cardiac disease in children, with quantitation largely based on nomograms. Over the past decade, significant efforts have been made to address the numerical and methodological limitations of earlier nomograms. As a result, robust and reliable pediatric echocardiographic nomograms are now available for most two-dimensional anatomical measurements, three-dimensional volumes, and strain parameters. These more recent nomograms are based on adequate sample sizes, strict inclusion and exclusion criteria, and rigorous statistical methodologies. They have demonstrated good reproducibility with minimal differences across different authors, establishing them as reliable diagnostic tools. Despite these advances, some limitations persist. Certain ethnic groups remain underrepresented, and data for preterm and low-weight infants are still limited. Most existing nomograms are derived from European and North American populations, with sparse data from Asia and very limited data from Africa and South America. Nomograms for preterm and low-weight infants are few and cover only selected cardiac structures. Although diastolic parameter nomograms are available, the data remain heterogeneous due to challenges in normalizing functional parameters according to age and body size. The accessibility of current nomograms has greatly improved with the development of online calculators and mobile applications. Ideally, integration of nomograms into echocardiographic machines and reporting systems should be pursued. Future studies are needed to develop broader, more comprehensive, and multi-ethnic nomograms, with better representation of preterm and low-weight populations, and to validate new parameters derived from emerging three- and four-dimensional echocardiographic techniques.

## 1. Introduction

Echocardiography is the first imaging modality for the assessment of congenital and acquired cardiac diseases at all ages, and quantification is an essential part of this modality [1,2]. In the pediatric age, as for any other body size measures, cardiac measures need to be compared to standards of normality according to age and body size [1,2,3,4]. Thus, pediatric echocardiographic nomograms are essential tools to understand whether a given cardiac measure of a given child with a given age, weight, and height is within the range of normality or not, and if not, how far it is from normality [1,2,3,4]. This process is essential to discriminate between healthy and pathological and to grade disease severity. Within the last 15 years, there has been a great amount of attention on pediatric echocardiographic nomograms, and great advances have been made [2,3,4,5,6,7,8,9,10,11,12,13,14,15,16,17,18,19]. Thanks to this improvement pediatric echocardiographic nomograms for most two-dimensional [2,3,4,5,6,7,8,9,10,11,12] and functional [13,14,15,16,17,18,19,20,21,22,23,24,25,26,27,28,29,30,31,32] parameters are currently available. Pediatric echocardiographic nomograms on myocardial strain analysis [33,34,35,36,37,38,39,40,41,42,43,44,45,46,47,48,49,50,51,52,53,54,55,56,57], 3D [58,59,60,61,62,63,64], and newer myocardial work parameters [65,66,67,68,69,70,71] are also recently becoming available. Despite advances, however, nomograms are not perfect tools, and limitations persist [72]. Despite advances in electronic tools [73,74,75], many current nomograms are still difficult to find and not present in online z-score calculators (e.g., parameterz.com) [74]. A few nomograms furthermore still present numerical and methodical limitations that may hamper their clinical utility [72]. Knowledge of the existence of current nomograms is essential for their use, and knowledge of their strengths and limitations is essential for their correct utilization [72]. 

The aim of the present study is to provide an updated and systematic evaluation of the reliability, comprehensiveness, and accessibility of pediatric echocardiographic nomograms published over the past two decades.

### 1.1. Literature Search Criteria

In April 2025, a research review was conducted utilizing three medical search engines: the National Library of Medicine, Science Direct, and the Cochrane Library. This review focused on Medical Subject Headings (MeSH) and the free-text terms “echocardiography”, and “normal values in children”.

The search parameters were further refined by incorporating the keywords “nomograms, Z-scores, neonates and infants, preterm, low-weight, three-dimensional, speckle tracking echocardiography, ventricular strain, atrial strain, diastolic”. Additionally, we identified other potentially relevant publications through a manual examination of references from all eligible studies and review articles, as well as the Science Citation Index Expanded available on Web of Science. The titles and abstracts of all articles identified through this search strategy were thoroughly evaluated. Manuscripts were excluded if they (a) utilized imaging techniques that differed from echocardiography, (b) contained a mixed population of adults and children, or (c) were written in a language other than English. This review was executed by the PRISMA 2020 statement [76].

All articles were evaluated independently by two specialists in pediatric echocardiography (M.C. and P.M.), and they were included in this study after reaching a consensus.

### 1.2. Search Results

**Figure 1:**
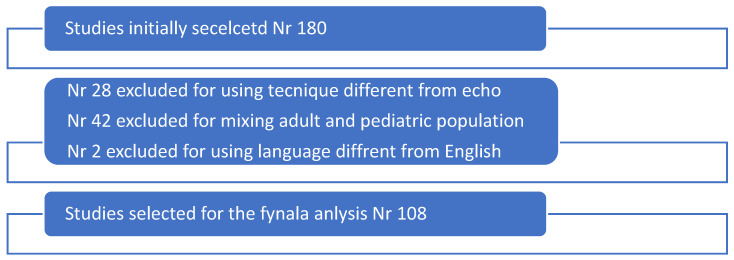
Selection diagram according to PRISMA guidelines.

Out of the 180 publications identified for potential inclusion in this study, 72 (40%) were excluded based on the above criteria, while 108 (60%) were ultimately selected for analysis and systematic review (Figure 1).

## 2. General Aspects

**1.**  **Accuracy of a nomogram**

Ideally, before trusting the use of a specific nomogram, one should verify its accuracy [2,72]. Accurate nomograms should be calculated by using the following: (i) the standardized method for cardiac measurements by echocardiography, (ii) a healthy population (with clear definition of inclusion and exclusion criteria), (iii) adequate sample size, (iv) consistent methods for standardization and expression of data, (v) rigorous statistical approach, and (vi) attention to confounders [2,72].

**(a)**  **Measurement Standardization**

The establishment of pediatric echocardiographic nomograms necessitates addressing multiple methodological issues. Foremost is the standardization of measurement techniques. Older nomograms presented inconsistencies regarding the timing of measurements within the cardiac cycle and the anatomical landmarks employed [2,72]. This generated confusion in the range of values proposed and difficulties in comparison among different nomogram sources. Guidelines for two-dimensional measurements in pediatric echocardiography were published in 2010 and recently renewed [1,7]. Protocols for imaging acquisition and basic measurements calculation have also been standardized for myocardial strain speckle tracking echocardiography and three-dimensional echocardiography [1,56,57,77,78]. These documents allowed a more standardized way to measure [1,56,57,77,78]. As a consequence, more recent nomograms have been calculated by using uniform methods of measurement, thus allowing comparison among them.

**(b)**  **Inclusion and Exclusion Criteria**

The careful definition of inclusion and exclusion criteria is essential to ensure the validity of normative datasets [2,72]. Only healthy pediatric subjects should be enrolled; however, the term “healthy” lacks a uniform operational definition and may differ across studies [2,78,79]. Recent investigations have generally converged on the exclusion of individuals with genetic syndromes, neuromuscular disorders, systemic or pulmonary hypertension, arrhythmias, connective tissue diseases, a family history of congenital heart disease, or suboptimal image quality [2,72]. Conversely, neonates with minor cardiac anomalies—such as a small patent ductus arteriosus or a restrictive patent foramen ovale in the early postnatal period—are frequently included [2,72]. Unresolved issues remain regarding the inclusion of preterm infants and the treatment of data from obese children [2,72].

**(c)**  **Sample Size Considerations**

The determination of appropriate sample size is a critical methodological component [2,72]. Contemporary guidelines recommend stratifying the pediatric population into six age groups [80,81,82] and enrolling a minimum of 100 subjects per group to achieve a 95% confidence interval with a margin of error of ±0.1, assuming normal distribution and a standard deviation of 0.5 [2,72]. Some authors advocate for at least 120 subjects per group, whereas others suggest a lower limit of 80 [2,72]. Given the reality that not all echocardiographic studies will yield analyzable data for every parameter, a pragmatic approach is to account for a 70–80% data completion rate [2,72]. Accordingly, to meet statistical requirements, each age group should include approximately 140 subjects [2,72]. Furthermore, to allow for sex- and ethnicity-specific analyses, these numbers should be doubled and then multiplied by the number of ethnic groups studied [2,72]. For six age groups, this equates to 840 subjects per sex, 1680 for both sexes, and up to 5040 individuals when considering three ethnic categories [2,72]. Nonetheless, most existing nomograms fail to meet these thresholds, which may limit their generalizability [2,72].

**(d)**  **Normalization and Expression of Data**

Normalization of echocardiographic variables relative to body size is fundamental [2,72,73]. Among the various body size metrics, body surface area (BSA), particularly when calculated using the Haycock formula [79], is the most widely adopted [2,72]. However, age, weight, height, heart rate, stroke volume, and left ventricular dimensions have also been used, particularly for diastolic and strain parameters [33,72]. The choice of indexing variable remains controversial, especially in the context of obesity, where BSA may obscure the influence of excess adiposity [79,83]. For left ventricular mass (LVM), indexing to height raised to an exponential power (most commonly height^2.7) is considered standard due to its superior approximation of lean body mass [79,84]. This approach has been endorsed by major guidelines and used to define left ventricular hypertrophy (LVH) in pediatric populations. However, age-specific considerations may be necessary, as fixed cut-offs are not universally applicable to younger children [79,83]. Recent findings suggest that indexing LVM to height^2.16 may reduce false positives in younger cohorts [79,83]. Additionally, bias introduced by body mass index (BMI) has been reported in Z-score models that rely on a single normalization variable [79,83]. Multivariable models incorporating both weight and height may mitigate this effect and improve the robustness of normative data [79,84].

**(e)**  **Statistical Modeling and Z-Score Calculation**

Z-scores are the most employed method for expressing normalized echocardiographic data [2,72]. Various regression models—including linear, polynomial, exponential, logarithmic, and square root functions—are used to generate Z-score equations [2,72]. The Lambda–Mu–Sigma (LMS) method has recently been utilized [9,85]. Despite its importance, heteroscedasticity—the non-constant variance of residuals—remains an underreported issue in many nomographic studies [86,87,88,89]. Residual distribution and mean square error (MSE) should be routinely evaluated to ensure model validity [2,72,83,86,87,88]. The model with the highest coefficient of determination (R^2^) that also satisfies assumptions of homoscedasticity is preferred [2,72]. There is no universally accepted R^2^ threshold, although values > 0.75 are generally considered substantial, and >0.6 acceptable [2,72]. In structural measurements, R^2^ values typically range from 0.5 to 0.9, whereas functional parameters show weaker correlations with body size, often with R^2^ values < 0.5 [2,72]. In such cases, descriptive statistics (mean ± standard deviation) or centile curves may serve as alternative approaches [72].

**(f)**  **Confounding Factors and Inherent Limitations**

The influence of confounding variables—including sex, ethnicity, prematurity, mode of delivery, and intra/inter-observer variability—must be carefully considered [2,72]. In neonates and infants, rapid physiological changes and growth during the first months of life can significantly affect echocardiographic measurements [2,72]. These dynamic changes, combined with external factors such as patient cooperation and operator expertise, may introduce unpredictable bias [2,72]. Certain imaging planes, such as apical four- and two-chamber views, are particularly susceptible to variability from minor probe angulation. Despite attempts at standardization, operator-dependent variability remains a significant limitation in echocardiographic quantification [2,72].

**2.**  **Nomograms for specific cardiac measurements**

**a.**  **Two-Dimensional Measurements**

Over the last decade comprehensive two-dimensional pediatric echocardiographic nomograms covering a broad range of parameters across valvular, arterial, chamber, and coronary artery dimensions have been published [2,3,4,5,6,7]. These nomograms cover the whole pediatric age, including neonatal age, and are characterized by a rigorous statistical approach [2,3,4,5]. All data were normalized by BSA and expressed with Z-score, obtained with coefficient of determination > 0.66 [3,4,10,12,18,90,91], with limited exceptions [6,11]. Current nomograms, including more recent ones, mostly derive from North America and Europe, while data from Asia [6,7] and Africa and South America [92,93] are limited. Echocardiographic nomograms by Lopez et al. (2020) [2] were developed from a large multi-ethnic cohort of 3566 healthy American children aged 0–18 years, including Caucasian, Black, and Hispanic subjects. These nomograms encompass a broad range of echocardiographic parameters (e.g., coronary arteries, valve areas, and ventricular volumes). Cantinotti et al. [3,4] proposed updated nomograms based on a homogeneous population of 1151 healthy Caucasian Italian children aged 0–18 years. These nomograms were derived from prospectively collected data at a single center with standardized imaging protocols and centralized analysis, improving internal consistency and reproducibility [3,4]. They include measurements of atrioventricular valves, the aorta (at multiple levels), pulmonary artery and branches, aortic arch segments, and left ventricular dimensions by M-mode [3,4]. 

**Figure 2:**
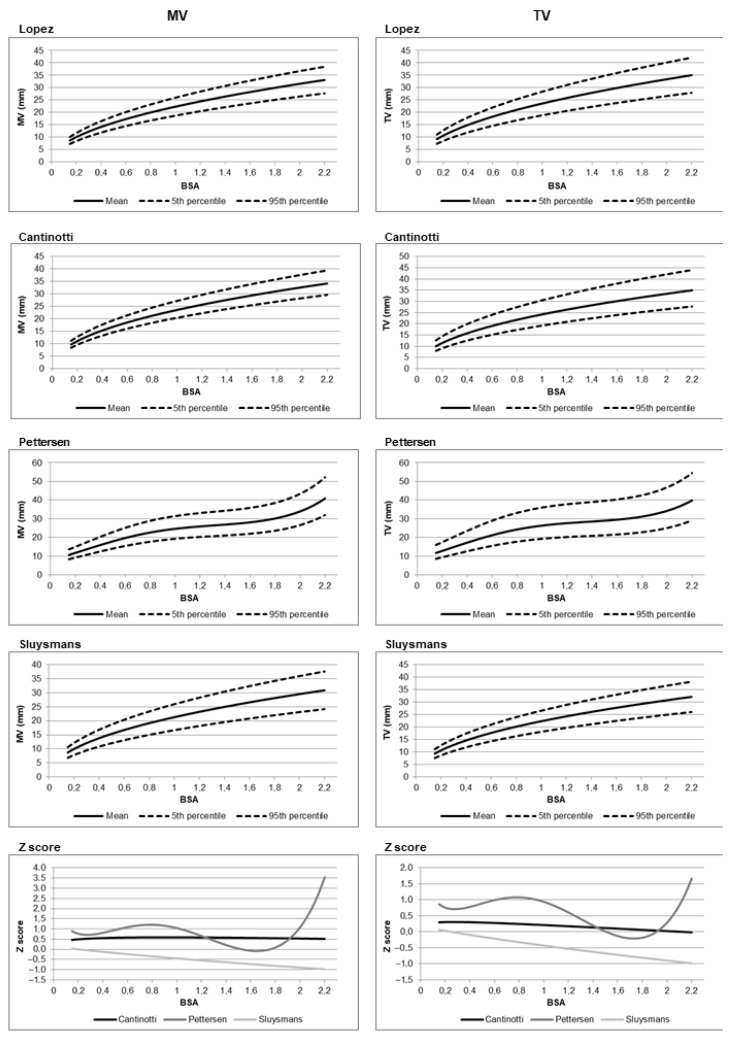
Percentile charts of predicted values for the mitral valve (MV) and tricuspid valve (TV) are shown for the nomograms by Lopez et al. [2], Cantinotti et al. [96], Pettersen et al. [91], and Sluysmans et al [90]. In addition, Z-score-based comparisons of predicted values from Cantinotti et al. and Pettersen et al. versus those from Lopez et al. (used as the reference standard) are presented. For example, with body surface area as the independent variable, the graphs illustrate the Z-score deviations of the mean predicted values from Cantinotti and Pettersen relative to the mean values reported by Lopez et al. Z-score calculation is performed as follows: Z-score (Cantinotti) = (predicted mean from Cantinotti et al.—predicted mean from Lopez et al.)/root mean square error (RMSE) of Lopez et al. Z-score (Pettersen) = (predicted mean from Pettersen et al.—predicted mean from Lopez et al.)/RMSE of Lopez et al.

Comparison among nomograms [3,4,72,90,91,94,95] revealed that the nomograms by Cantinotti [3,4] and Lopez [2] were the most statistically similar, particularly across a wide range of body surface areas (BSAs). Z-score differences between these two datasets were mostly below 0.5, suggesting limited clinical discordance. In contrast, older nomograms by Sluysmans and Colan and Pettersen et al. [90,91], based on smaller or less diverse cohorts, showed greater divergence from Lopez et al., particularly at the extremes of BSA. Figure 2 illustrates an example of the differences between older and more recent nomograms. Percentile curves for mitral and tricuspid valve dimensions are shown, along with a comparison of predicted values expressed as Z-scores, using the nomograms by Lopez et al. [2] as the reference standard. For a few specific parameters such as aortic isthmus and ascending aorta diameters, however, even among nomograms by Lopez and Cantinotti [2,3,4], significant differences were noted. For example, at a BSA of 0.21 m^2^, the aortic isthmus diameter was significantly smaller in Cantinotti’s nomograms [3,4] (mean 4.47 mm) compared to Lopez’s (mean 5.73 mm) [2], with a Z-score difference of −0.70. These discrepancies, especially in the infant population, may result in clinically relevant differences in diagnosis and management of congenital heart disease, such as the classification of valve hypoplasia or aortic arch hypoplasia.

Also more recent nomograms, furthermore, present a few limitations. While the Lopez [2] nomograms benefit from a large, multi-ethnic sample, they also suffer from methodological limitations, such as retrospective data collection from 19 centers and broad inclusion criteria for ethnic subgroups, potentially reducing statistical power to detect meaningful inter-ethnic differences. In contrast, Cantinotti et al.’s nomograms [3,4] offer higher standardization but are limited in ethnic diversity.

Despite more recent nomograms covering a broad range of 2D parameters, gaps for a few measurements remain. Relatively limited data are present for a few cardiac structures including cardiac chamber areas in apical four- and two-chamber views [96], left ventricular volume using subxiphoid imaging [2], left atrial volume via the biplane area-length method [91], and right ventricular dimension using sub-xyphoid view [20].

In Table 1 major pediatric echocardiographic nomograms for 2D parameters are summarized, while, in Table 2, major pediatric echocardiographic nomograms for coronary arteries are reported.

**b.**  **Diastolic Function and Other Functional Parameters**

Assessment of diastolic function is critical in the management of both congenital and acquired heart disease in children [13,14,15,16,17,18,19,20,21,97]. However, the interpretation of diastolic indices remains challenging, especially in neonates and infants, due to the rapid maturational changes that occur in early life [13,14,15,16,17,18,19,20,21,97]. Age-specific normative values are essential to differentiate physiological variation (such as the common inversion of mitral E and A wave velocities in neonates) from pathological findings [13,14,15,16,17,18,19,20,21].

Numerous nomograms for pulsed and tissue Doppler-derived diastolic parameters have been published [13,14,15,16,17,18,19,20,21], but many suffer from methodological limitations, including heterogeneous measurement techniques and inconsistent age stratification [94]. The scarce correlation of diastolic parameters with most body size parameters and age hampered the possibility of performing z-score equations with sufficient statistical power [95]. Thus, most studies are limited to providing data as percentiles or mean ± standard deviation [95]. Compared to Z-scores, the use of percentiles or mean values is limited in assessing the severity of cardiac abnormalities in pediatric echocardiography. Percentiles only indicate a relative rank without quantifying deviation from normal, while mean values do not account for individual variability [2,72]. Z-scores offer a standardized, continuous measure of how far a value deviates from the mean, adjusting for body size and age [2,72]. This enables a more precise assessment of abnormality severity and supports clinical decision making more effectively [2,72].

Recent investigations [14,17,18] have sought to overcome these issues. Dallaire et al. reported z-scores for a broad set of diastolic indices derived from pulsed, tissue, and color Doppler in a cohort of 233 healthy children aged 1–18 years [14]. However, the majority of indices demonstrated a nonlinear relationship with somatic growth and significant heteroscedasticity, and the correlation coefficients (R^2^), although not reported, appeared low based on the scatterplots. Additionally, although heart rate was found to influence some parameters, it was not incorporated into the final analysis.

Roberson et al. [17] provided normative data on tissue Doppler-derived mitral and tricuspid velocities from 634 healthy children aged 1 day to 18 years, normalized by age and heart rate. However, z-scores were only provided for a limited subset of variables, and regression coefficients (R^2^) were not reported. The most extensive study to date, involving 904 healthy Italian subjects aged 0–17 years [18], reported very weak correlations (R^2^ ranging from 0.18 to 0.53) between mitral Doppler indices and body surface area, and no significant associations with heart rate or blood pressure. Due to the low R^2^ values, z-score computation was not feasible, and normative data were presented as percentiles and mean ± standard deviation stratified by age and body surface area [18].

Despite these limitations, recent nomograms present reproducible patterns that can assist clinicians in routine practice. Normal ranges published in recent studies are consistent in older children and comparable to adult reference values [14,17,18]. Nonetheless, discrepancies remain. For instance, the late diastolic flow velocity or A wave at the mitral valve for a 3-year-old child varies from 42 cm/s [13] to 50 cm/s [19] to 61 cm/s [16], depending on the nomogram used.

Data on neonates and infants remain sparse and inconsistent, complicating classification of diastolic dysfunction in this age group [94]. Adult standards may be applicable in children over 3 years old but are unreliable in younger children, especially due to high neonatal heart rates [1,95].

In Table 3 and Supplemental Table S1 major pediatric echocardiographic nomograms for functional parameters are reported.

**c.**  **Strain Analysis**

Non-invasive evaluation of left ventricular myocardial strain and strain rate is gaining widespread acceptance for the follow-up and management of children with congenital heart disease (CHD) [33,34,35,36,37,38,39,40,41,42,43,44,45,46,47,48,49,50,51,52,53,54,55]. Over the past decade, several nomograms for left ventricular strain and strain rate in the pediatric population have been proposed [33,34,35,36,37,38,39,40,41,42,43,44,45,46,47,48,49,50,51,52,53,54,55]. As with other functional parameters, correlations of speckle tracking echocardiography (STE) values with body size parameters and age were weak; thus, most studies normalized values by age [9,21,33,34,35,36,37,40,42,43,44,45,47,52], reporting data as means with standard deviations [9,33,35,40,42,43,44,45,47,50,52]. Although the heart rate was typically assessed, Boettler et al. [98] were the only group to apply formal normalization for heart rate. Dallaire et al. [46] proposed z-score equations normalized by body surface area (BSA), but the correlation between strain parameters and BSA was extremely weak (R^2^ ranging from 0.03 to 0.002), introducing substantial bias in the resulting equations [46].

Some of these limitations may be partially overcome. For instance, three-dimensional echocardiography offers potential advantages by enabling more comprehensive spatial assessment and avoiding loss of speckle due to out-of-plane motion [41]. Similarly, newer studies have begun to implement more standardized methodologies and wider sample cohorts [47,52]. Nevertheless, certain intrinsic limitations—such as high heart rate and rapid physiological changes—make strain analysis particularly unreliable in neonates and infants [99]. Beyond infancy (age > 1 year), strain values tend to be more stable and reproducible across age groups [100]. For example, in a 3-year-old boy, global longitudinal strain varied from −23.6% [45] to −20.7% [44] to −21.3% [101], while, in a 14-year-old girl, reported values ranged from −21.8% [45] and −21.8% [44] to −22.7% [99].

Due to the relatively small variation in strain values with age, two recent meta-analyses proposed a single reference range for pediatric left and right ventricular strain [l49]. However, this approach oversimplifies the problem and runs counter to the foundational principle of nomograms [72,73,100]. Larger, adequately stratified studies are needed to clarify maturational trends in strain parameters across the pediatric age spectrum [72,73,100].

Data on basal–apical rotation, torsion, twist, and untwist in children remain limited [100,101,102]. Kim et al. [102], using 2D speckle tracking echocardiography in 80 children (3 months–15 years), found that both apical and basal rotation and twist (when corrected for left ventricular length) decreased with age, whereas untwisting recoil was unaffected by age. Takahashi et al. [102], in a larger cohort (111 subjects, aged 3–40 years), similarly demonstrated that twist, untwist, and deformation rates were higher in younger individuals. Notably, most of these parameters correlated more strongly with heart rate than with age [102]. A 3DSTE study on 311 subjects from childhood to adulthood reported the lowest torsion values in children and adolescents, with progressive increases reaching a plateau in adulthood [103].

**(c1) Ventricular Strain**

*a. Relationship with Age and Body Size*

Several studies have demonstrated a mild inverse correlation between strain parameters and age, with correlation coefficients ranging from −0.8 to −0.007 [99]. However, these relationships are nonlinear, as shown in large pediatric cohorts [99]. For example, left ventricle global longitudinal strain (LV GLS) values are higher in infants and toddlers (31 days–24 months) compared to older age groups, while significantly lower values are observed in adolescents (11–18 years) [99]. In contrast, LV circumferential strain (CS) shows minimal variation across age groups, with slightly higher values in older children [98]. No significant age-related differences were found for right ventricle (RV) GLS [104].

In a cohort of over 1000 subjects under 21 years, both LV longitudinal and circumferential strain peaked around 4–5 years of age and declined thereafter [52]. Similarly, RV longitudinal strain followed a similar pattern [52]. Mild inverse correlations were also observed between strain values and body surface area (BSA), although these were generally weak [52].

*b. Sex Differences*

Most studies reported no significant sex-related differences in strain parameters [100]. When present, differences were minimal and primarily observed in adolescents, likely due to pubertal timing differences [99]. Some data showed slightly higher LV and RV strain values in females, though not consistently across studies [104].

*c. Inter-Vendor Variability*

Vendor-related variability is a known limitation in STE [53,54,55]. Good agreement was found between GE and TomTec platforms for LV GLS, with intraclass correlation coefficients (ICCs) ranging from 0.88 to 0.9 [53,54,55]. Variability, however, increased for LV CS and in children under 3 years, where ICCs were notably lower. Comparisons between Philips QLAB (versions 10.5, 10.8, AutoSTRAIN) and TomTec software version TTA2.00.03 revealed significant discrepancies in strain values, especially for older QLAB versions [53,54,55].

Agreement between GE and Philips platforms was poor, with interclass correlation coefficients (ICCs) as low as 0.34 for longitudinal and 0.12 for circumferential strain. Intra-vendor software version differences were also notable; QLAB 10.5 tended to yield higher strain values than QLAB 10.8 and AutoSTRAIN, especially in older children [53,54,55].

*d. Inter- and Intra-Observer Variability*

Observer variability depends on both the software and frame rate. For Philips QLAB 10.8, intra-observer agreement for LV strain was moderate (ICC: 0.5–0.79), while inter-observer agreement ranged from mild to good (ICC: 0.8–0.96) [53,54,55,99]. AutoSTRAIN showed excellent intra- and inter-observer agreement, especially for LV strain [53,54,55,98]. TomTec and GE also demonstrated good reproducibility, with ICCs > 0.85 for LV GLS and slightly lower values for CS. Higher frame rates were associated with better reproducibility [53,54,55,99].

**(c2) Atrial Strain**

*a. Maturational Variation*

Atrial strain parameters show significant age-dependent variations [33,36,43,49,50]. Nomograms based on 2D speckle-tracking echocardiography (STE) demonstrate a nonlinear positive correlation between left atrial (LA) reservoir strain (LASr) and age, as well as a nonlinear negative correlation between LA contractile strain and age, with the most rapid changes occurring in infancy [33,36,43,49,50]. Both LA and right atrial (RA) conduit strain values are reduced in younger children, whereas contractile strain values are higher during early life [33,36,43,49,50].

Using 3D STE [49], all LA strain components show a declining trend with age, although the correlations are weak (r = 0.14 for global longitudinal strain; r = 0.31 for global 3D strain). 

*b. P-Gating* vs. *R-Gating and Software Differences*

Strain values derived from P-wave gating are consistently lower than those from R-wave gating for both LA and RA, across all pediatric age groups [33,100]. Furthermore, significant differences are observed between atrial strain values obtained using atrial-specific software (QLAB 10) versus older ventricular-specific software (QLAB 9) adapted for atrial analysis [33,98].

In particular, LASr values are significantly lower when assessed with atrial-specific software across all age groups (*p* < 0.001), while RA Sr values differ only in certain age groups. LA conduit strain (LASct) values are higher in younger children and lower in older ones when measured with QLAB 10 compared to QLAB 9 [33,99].

In Table 4 and Table 5, major pediatric echocardiographic nomograms for STE strain parameters are reported.

**d.**  **Three-Dimensional Measurements**

The potential of three-dimensional (3D) echocardiography for the diagnosis and management of CHD—particularly valvular abnormalities—has been increasingly recognized [80]. Compared to 2D echocardiography, 3D imaging provides superior accuracy in assessing valve areas and chamber volumes, as well as a more realistic evaluation of right ventricular size [80]. However, pediatric data remain limited [58,59,60,61,62,63,64]. Aside from functional parameters [49,58], few nomograms exist for 3D-derived anatomical measures, and they all pertain to ventricular [58,59,60,61,62] or atrial volumes [64]. Either Z-score [49,58,59,62] has been used for normalization, and R2 when reported [49,62] were good (e.g., ≥0.79). The Finnish group has published a few studies based on a cohort of 169 healthy subjects aged 2–27 years, including data on atrial and ventricular function, aortic and mitral annular areas, and left ventricular mass [103,105,106]. These studies showed that 3D echocardiography yielded larger valvular area estimates than 2D and Doppler-based methods [106], while left ventricular mass calculated by 3D methods was lower than estimates from 2D and M-mode echocardiography [103]. 

In Table 6 major pediatric echocardiographic nomograms for 3D parameters are reported.

## 3. Limitations of Current Nomograms

Despite the availability of pediatric echocardiographic nomograms covering a broad range of parameters and age groups, significant gaps in knowledge remain. Data on premature and low-birth-weight infants are still limited [107,108,109,110,111,112,113], although this population presents unique challenges for clinical decision making due to differences in cardiopulmonary transition and cardiac development compared to term and normal-weight neonates. Consequently, the application of nomograms derived from term, normal-weight infants to this subgroup may lead to suboptimal assessments.

The limited data available for preterm infants primarily include measurements of left ventricular diameters and wall thickness [107,108,109,110,111,112], as well as aortic and left atrial dimensions assessed by M-mode [107,108,109,110,111,112,113]. Only a few studies have reported M-mode data on right ventricular end-diastolic diameter [112], pulmonary artery dimensions [112], and Doppler-derived velocities of the valves and great vessels, such as the aorta and pulmonary artery [111].

Most currently available nomograms originate from Europe and North America, while data from Asia are scarce [6,7,9,11,107,109,112], and those from Africa [38,42,89,92] and South America [112] are extremely limited. Interestingly, among the limited neonatal datasets available, only a few originate from Europe [111] and North America [113], with the majority coming from Asia [107,108,109,112] and Australia [110]. Table 7 summarizes the main pediatric echocardiographic nomograms currently available for preterm and low-birth-weight infants.

Data for Junior Athletes is also extremely limited [114,115,116,117,118] (Supplemental Table S2). Nomograms calculated in the general population cannot be applied to athletes, whose cardiac dimensions are physiologically increased. Lastly, a few CHD-specific nomograms are lacking. 

## 4. Future Directions

As echocardiography continues to evolve—particularly with the emergence of advanced functional and 3D modalities—nomograms must also be updated to reflect these developments [72]. New parameters are continually being introduced, requiring the creation of new normal reference ranges. Ideally, nomograms should be easily accessible to clinicians and integrated into digital platforms. Free online z-score calculators (e.g., parameterz.com, CardioZ, BabyNorm Calculator) allow for the combination of multiple nomograms and help clinicians assess whether measurements fall within normal limits [73,74,75]. While there is sufficient coverage for 2D measures, not all data, however, are currently available on these tools [73,74,75]. Unfortunately, new normative data deriving from 3D and strain analysis remain difficult to find, as well as the ranges of normality for most of the 2D functional parameters. Artificial intelligence (AI) [119,120] may facilitate a more efficient, comprehensive, and rapid application of available Z-scores, enabling easier comparison across different datasets. Furthermore, AI could support the identification of strengths and limitations inherent to the specific Z-score references employed.

The integration of z-scores into echocardiographic reporting software will be a crucial step toward standardization and clinical utility. Ultimately, embedding nomogram-based tools directly into echocardiographic machines could enable the real-time detection of abnormal values. Furthermore, the development of software supporting automated or semi-automated measurements will help reduce inter- and intra-observer variability [72].

Collaborative development with echocardiographic vendors aimed at integrating AI-based tools into ultrasound systems for semi-automated measurements and automatic Z-score computation would offer substantial clinical benefits.

Multicenter collaborations and the establishment of standardized data-sharing platforms are strongly recommended to overcome current data gaps in specific subpopulations, such as preterm and low-birth-weight neonates, junior athletes, and individuals from African, South American, and Asian regions.

## 5. Conclusions

The use of nomograms is strongly recommended for quantitative pediatric echocardiography. Although earlier nomograms were limited by methodological and numerical flaws [72], recent efforts have led to substantial improvements and the development of more robust tools. While reliable nomograms now exist for many 2D anatomical measurements, 3D volumes, and strain parameters, data for diastolic parameters remain heterogeneous due to the difficulty in normalizing these data by age and body size parameters. Future works should focus on comparison among different ethnic groups, including African and South American (which are currently lacking), and on premature low-weight babies, whose data are currently lacking.

## Figures and Tables

**Table 1:** Major pediatric echocardiographic nomograms for 2D measures.

Author	Parameters Evaluated	Sample Size	Normalization	Data Expression	R2
Pettersen MD USA 2008 [91]	Aortic arch at 3 levels, Abd Ao, pulmonary annulus, MPA, RPA, LPA, aortic annulus, Ao root, Asc Ao, (PLA), MV and TV annulus; LV M-mode	n. 748 0–18 yrs	BSA NR	Z-score	0.992–0604
Zilberman MV USA 2005 [94]	Annulus of aorta, pulmonary, MV, TV	N. 748 0–18 yrs	BSA DuBois	Z-score	0.91–0.70
Gautier M USA 2010 [95]	Ao annulus, Ao root, STJ, Asc. Ao	n. 353 2–18 yrs	BSA Dubois	Z-score	NR
Cantinotti M Italy 2014 [3]	Aortic arch (IA-LCA, LCA-LSA, after LSA, isthmus), thoracic aorta, Ao annulus, Ao root, STJ; Asc. Ao, aorta at the diagram, pulmonary annulus, MPA, RPA, LPA, IVC, LV M-mode	n. 445 0–36 months	BSA Haycock	Z-score	0.89–0.49
Cantinotti M Italy 2014 [96]	LV, diameters and area in 4 and 2 ch views, 4 ch RV, RA, LA diameters and areas	n. 1091 0–17 yrs	BSA Haycock	Z-score	0.96–0.86
Cantinotti M Italy 2017 [4]	Aortic arch (IA-LCA, LCA-LSA, after LSA, isthmus), thoracic aorta, Ao annulus, Ao root, STJ; Asc Ao, aorta at the diagram, pulmonary annulus, MPA, RPA, LPA, IVC, LV M-mode	n. 1151 0–17 yrs	BSA Haycock	Z-score	0.93–0.85
Lopez L 2017 Multicenter USA [2]	Ao, PA, MV (AP and lat) and TV (Ap and lat) annulus, Asc Ao, STJ, Asc Ao, MPA; LPA; RPA, MVA, TVA, arch (prox, dist, isthmus), LVEDA (PSA), LVEDV (5/6 × LVEDA × LVEDL), LVEDL (4 ch). LVM.	n. 3215 0–18 yrs (35% whites, 31% black, 34% other)	BSA Haycock	Z-score	NA
Gokhroo RK 2017, India [6]	Ao annulus, Ao root, STJ, Asc Ao, MVA, TVA. LA and RA diameters, RV basal, and mid-cavity diameter, RV length	n. 746 4–15 yrs	BSA Haycock	Z-score	0.35–0.114

Ao = aorta; Abd= abdominal; Asc = ascending; AP = anterior–posterior; IA = innominate artery; lat = lateral; BSA = body surface area; IVC = inferior vena cava; LA = left atrium; LCA = left carotid artery; PA = left pulmonary artery; LSA = left subclavian artery; LV= left ventricle LVM = left ventricular mass; MPA = main pulmonary artery = mitral valve; NA = not applicable; NR = not reported; PA = pulmonary annulus; RA = right atrium; LVEV= left ventricle end diastolic volume; LVEDL = left ventricle end-diastolic length; LVEDA = left ventricle end-diastolic area; RPA = right pulmonary artery; STJ = sino-tubular junction; TV = tricuspid valve; TVA = tricuspid valve area; 4 ch = four chamber; yrs = years; and R2 = coefficient of determination.

**Table 2:** Major pediatric echocardiographic coronary artery nomograms.

Author	Sample Size	Coronary Artery	Normalization	Data Expression	R2
Kobayashi T 2016 Japan [9]	n. 3851 <18 yrs	RCA, LMCA, LCX, LAD	BSA Haycock	Z-score	NA
Dallaire F 2010 Canada [10]	n. 1033 Age NR	RCA, LMCA, LCX, LAD	BSA Haycock	Z-score	0.71–0.79
Zhang Y et al., 2014 China [11]	n. 506 1 day–18 yrs	RCA, LMCA, LCX, LAD	BSA Haycock	Z-score	0.50–0.51
Olivieri L, USA 2009 [12]	n. 432 0–20 yrs	LMCA, LAD, RCA	BSA DuBois	Z-score	0.79–0.84
Lopez L et al., 2017, USA [2]	n. 3215 0–18 yrs	LMCA, LAD, RCA	BSA Haycock	Z-score	NA
Cantinotti et al. 2016 Italy [18]	n. 606 0–18 yrs	LMCA, LAD, RCA, LCX Proximal LMCA and RCA	BSA Haycock	Z-score	0.6–0.628

LMCA = left main coronary artery; LAD = left descending artery; LCX = left circumflex artery; CA = right coronary artery; BSA = body surface area; and NA = not applicable.

**Table 3:** Major pediatric echocardiographic nomograms for functional diastolic parameters.

Author	Parameter Evaluated	Sample Size	Normalization	Data Expression
O’ Leary PW 1998 USA [13]	Pwd MV: E, A, E/A, EDT, IVRT	n. 223 3–17 yrs	Age Groups	Mean ± SD
Schmitz L. 2004 Germany [19]	Pwd MV: E, A, EDT, IVRT	n. 311 0–19 yrs	Age Groups	Mean ± SD
Eidem BW 2004 USA [15]	Pwd MV E, A, E/A Pwd TV: E, A, E/A TDI MV: e′, a′, E/e′ TDI TV: e′, a′, E/e′	n. 325 0–18 yrs	Age groups	Mean ± SD
Ciccone M. 2011 Italy [97]	Pwd MV E, A, E/A Pwd TV: E, A, E/A TDI MV: e′, a′, E/e′	n. 53 (neonates)	Term and Preterm	Mean ± SD
Dallaire F. 2015 Canada [10]	Pwd MV E, A, E/A, DT, TDI MV: e′, a′, E/e′,	n. 233 1–18 yrs	BSA (Haycock)	Z-scores
Cantinotti M. 2016 Italy [18]	Pwd MV E, A, E/A, DT TDI MV: e′, a′, E/e′	n. 904 0–17 yrs	Age and BSA (Haycock)	Percentiles
Roberson DA 2007 USA [17]	TDI MV: e′, a′, s′ TV s′	n. 634 0–18 yrs	Age, Heart rate	Z score charts only for e′, s′. TV s′ Z score equation not provided, R2 not provided
Cui W 2007 USA [16]	TDI, Pwd, M-mode: LV and RV Tei index	n. 289 0–18 yrs	Age groups	Mean ± SD

A = mitral valve pulsed Doppler A velocity; a′ = lateral mitral valve annulus TDI a velocity; BSA = body surface area; E = mitral valve pulsed Doppler E velocity; DT = deceleration time; EDT = mitral E deceleration time; IVRT = isovolumetric relaxation time; Pwd = power Doppler; LV = left ventricle; MV = mitral valve; RV = right ventricle; SD = standard deviation; TDI = tissue Doppler imaging; R2 = coefficient of determination; TV = tricuspid valve; s′ = septal valve annulus TDI s′ velocity; e′ = mitral valve annulus TDI e′ velocity; and SD = standard deviation.

**Table 4:** Major pediatric STE nomograms for ventricular strain values.

*Author*	*Population*	*Measures*	*Software*	*Data Norm*	*Data Expression*
Adar A 2019 USA [35]	N = 312 3 days–20.5 yrs	LV LS, CS, and synchrony;	Echo: Philips Epiq Software: QLAB v.10.5 (Philips)	Age groups	Mean, SD
Harrington JK 2021 USA [37]	N = 577 1–18 yrs	LV SR	Echo: Philips Software: QLAB 9.0 (Philips)	Age	Z-scores
Koopman LP 2019 The Netherlands [40]	N = 103 mean 10.8 yrs IQR 7.3–14.3 years.	LV LS, CS	Echo: Philips IE33 Software: QLAB 9.0 (Philips)	Age groups	Mean, SD Percentiles
Romanowicz J 2023 USA [52]	N = 1032 <21 years old	LV and RV LS, LV CS	Echo: Philips Epiq Software: Auto-strain, QLab 10.5 10.8	Age	Mean, SD Z-scores
Kamel H 2022 Egypt [9]	N = 200 3.832 ± 1.522 yrs range 0.1–5.9 yrs	LV GLS, GCS, GRS 2d and 3D	Echo: Vivid E9 (GE). Software: EchoPAC V113 (GE)	Age groups	Mean, SD
Kotby AA 2023, Egypt [42]	N = 250 1–16 yrs	LV LS	Echo: GE Software: EchoPAC	Age groups	Mean, SD
Aristizábal-Duque CH 2022 Spain [43]	N = 156 6 t- 17 yrs	LVGLS, RVGLS, RV free wall LS, LA	Echo: Philips IE33 Software: 13.0 of QLab 13 (Philips)	Age groups, BSA	Mean, SD
Klistisic L, 2013 The Netherlands [45]	N = 183 0–19 yrs	LV LS, CS, RS	Echo: Vivid 7 GE Software: EchoPAC GE	Age groups	Mean, SD
Marcus K, 2011 USA [44]	N = 144 0–19 yrs	LV LS, CS, RS	Echo: Vivid 7 GE Software: EchoPAC GE	Age groups	Mean, SD
Zhang L, 2013 China [41]	N = 226 0–18 yrs	LV 3D STE LS, CS, RS	Echo: Philips IE33 Software: TomTec TTA2.00.03	Age groups	Mean, SD
Cantinotti M, 2018 Italy [47]	N = 721 31 days–18 yrs	LV, LS, CS RV LS	Echo: Epiq/IE33 (Philips) Software: Q-LAB 9 Philips	Age groups, gender	Mean, SD
Dallaire F, 2016 Canada [46]	N = 233 1–18 yrs	LV LS, CS	Echo: Vivid 7 GE Software: EchoPAC GE 7	BSA	Z-scores
Acheampong B, 2023 USA [34]	N 142 0–18 yrs	LV LS, CS, RS	Echo: Philips and Siemens Software: Cardiac Performance Analysis version 3.0 *	Age groups	Percentiles

CS = circumferential strain; GCS = global circumferential strain; IQR = interquartile range; LA = left atrium; LV = left ventricle; LS = longitudinal strain; N = number; GLS = global longitudinal strain; GRS = global radial strain; RV = right ventricle; RS = radial strain; SD = standard deviation; and SR = strain rate. GE = General Electric Ultrasound, Horten, Norway; EPIQ platform = Philips Healthcare, Andover, Massachusetts; TomTec = TomTec Imaging Systems, Germany; Siemens Healthineers Erlangen, Germany, which integrates the TomTec auto-strain software; * Independent software.

**Table 5:** Major pediatric STE nomograms for atrial strain values.

*Author*	*Population*	*Measures*	*Software*	*Data Norm*	*Data Expression*
Cantinotti M, Italy [33]	N = 836 31 days–18 yrs	2D LA and RA strain	Echo: Epiq/IE33 (Philips) Software: Q-LAB, and Q- LAB 10 (Philips)	Age groups	Mean, SD
Ghelani S, 2013 USA [49] [21]	N = 196 4 days–20.9 yrs	3D LA volumes and strain	Echo: Philips IE33 Software: 4D LV Analysis, TomTec 3.1	Age	Z-scores
Kutty S 2013, USA [50]	N = 153 3–20 yrs	2D LA and RA strain	Echo: GE Vivid 7, Software: EchoPAC Bt11 GE	Age groups	Mean, SD
Jimbo S 2020 Japan [36]	N = 112 (median 12.0 years; range 6–16 years)	2D LA strain and SR	Echo: NR Software: TomTec	Age groups	z-scores
Aristizábal-Duque CH 2022 Spain [43]	N = 156 6 t- 17 yrs	2D LA strain	Echo: Philips IE33 Software: 13.0 of QLAB 13 (Philips) °	Age groups, BSA	Mean, SD

LA = left atrium; N = number; NR = not reported; RA = right atrium; SD = standard deviation; SR = strain rate; 2D = two-dimensional; 3D = three-dimensional; and SD = standard deviation ° dedicated to atria. Epiq, iE33 systems (Philips Medical Systems, Bothell, WA, USA), QLAB 9; Philips Medical Systems, Andover, MA, USA), QLAB 10; Philips Medical Systems, Andover, MA, USA, TomTec Imaging System Freisinger Str. 9, 85716 Unterschleissheim, Germany, Vivid E 9/95 GE = General Electric Health Care, Chicago, IL, USA.

**Table 6:** Major pediatric echocardiographic nomograms for 3D measures.

Author	Sample Size	Measures	Echo Machine	Software	Normalization
Kubler JD 2018, Boston USA [58]	n. 238 0.4–17.9 yrs	3D LV volumes, stress, and strain	Philips IE33 and Epiq	4D LV Analysis; TomTec 3.1	Z-score R2 NR
Jone PN 2021, Multicenter USA [59]	n. 698 0–18 yrs	3D LV volumes	GE Vivid E9/E95; IE33/EPIQ, Siemens SC2000,	4D LV Analysis, TomTec 4.0,	Z-score R2 NR
Krell K 2018 Multicenter Germany [60]	n. 370 1 day–219 months	3D LV volumes	IE33, Philips	QLab 9.0 (Philips) and TomTec 4DLV2.7	Percentiles
Cantinotti M 2019, Italy [62]	n. 800 118 yrs	3D LV volumes	IE33, Philips	QLab 9.0 (Philips)	Z score R2 0.83–0.84
Herberg U 2021 Multicenter Germany [61]	n. 545 1 day–216 months	3D RV Volumes	IE33, Philips or Vivid 7, GE)	VMS, Ventripoint	Percentiles
Ghelani S, 2017 USA [49]	n. 196 4 days-20.9 yrs	LA volumes and strain	Philips IE33	4D LV Analysis; TomTec 3.1	Z score R2 0.79–0.98
Linden K, 2019, Germany [64]	n. 432 0 days–22 months	3D LA volumes	Philips IE33 or Vivid E9/95) (GE)	4D LV Analysis; TomTec 3.1	Z score R2 NR
Poutnaen T, 2016, Finland [106]	n. 168 2–27 yrs	MVA, AVA	GE Vingmed System Five	GE	Mean SD
Poutanen T, 2003, Finland [103,105]	n.169 2-27 yrs	3D LV and LA volumes, LV mass	GE Vingmed System Five	GE	Mean SD

AVA = aortic valve area; LA = left atrium; LV = left ventricle; RV = right ventricle; 3D = three-dimensional; 4D = four-dimensional; R2 = coefficient of determination; NR = not reported; MVA = area; and SD = standard deviation. Epiq, iE33 systems (Philips Medical Systems, Bothell, WA, USA), QLAB 9; Philips Medical Systems, Andover, MA, USA), QLAB 10; Philips Medical Systems, Andover, MA, USA, VMS = Ventripoint diagnostic; 18 Hook Ave, Unit 101, Toronto, Ontario M6P 1T4 Canada. TomTec Imaging System Freisinger Str. 9, 85716 Unterschleissheim, Germany, Vivid E 9/95. GE = General Electric Health Care, Chicago, Illinois, US; Siemens Healthineers, Erlangen, Germany, GE Vingmed System Five, Horten, Norway.

**Table 7:** Major pediatric echocardiographic nomograms for preterm low-weight babies are reported.

Author	Population	Parameters Evaluated	Normalized By	Data Expression
Lu DF 2022, China [107]	489, 264 M GA 32 (24–36.7) weeks BW 1700 (650–3180) g BSA 0.13 (0.07–0.20) m^2^	M-mode: LV, LA, Ao, MV A, E, E/A	GA BW BSA	Percentiles R 0.07–0.616
Wang S, 2022 China [109]	1570 term and preterm	M-mode: LV, LA, Ao,	GA BW BSA L	Percentiles
Calado C, 2021 Australia [110]	1244 Preterm infants ≤32 weeks ≤ 1500 g GA 27.3 ± 2.2 (22–32), weeks Age 2.2 ± 1.5 (1–7) days BW 999 ± 266.9 (349–1500) g BSA 0.10 ± 0.02 (0.05–0.14) m^2^	M-mode: LV, LA, Ao, RVED	BW	Mean SD Percentiles
Abushaban L 2017 Kuwait [112]	268, 26 M GA 29.8 (±2.38) weeks BW 1479 (±413 SD) g BSA 0.123 (0.07 to 0.19) m^2^	MPA, LPA, RPA	BSA	Z-score, mean SD, R not reported, scatterplot indicates low R2
Choudry S,2017, USA [113]	503 < 2 kg BW 1.2 ± 0.74 kg GA 21.29 ± 22.56 BSA 0.11 ± 0.03	M-mode: LV	Weight, L, BSA	Percentiles Z-score R2 NR
Skelton R 1998 UK [111]	79 < 34 weeks BW 500–2499 g GW 23–33 weeks	M-mode: LV, Ao Doppler velocity: Asc Ao, Desc Ao, PA, MV, TV	BW, GA	Mean, range

Asc Ao = ascending aorta; Desc AO = descending Aorta; BSA = body surface area; BW = body weight; GA = gestational age; L = length; LA = left atrium; LV = left ventricle; MV = mitral valve; peak mitral valve flow rate E; peak mitral valve flow rate A; LPA = left pulmonary artery; MPA = main pulmonary artery; MV = mitral valve; PA = pulmonary artery; RPA = right pulmonary artery; RVED = right ventricle end diastolic diameter; TV = tricuspid valve; R2= coefficient of determination; and NR = not reported.

## Data Availability

The data presented in this study are available upon request from the corresponding author.

## Supplementary Materials

The following supporting information can be downloaded at https://www.mdpi.com/article/10.3390/jcm14155215/s1, Supplemental Table S1: Major pediatric nomograms for functional assessment of the right ventricle by echocardiography; Supplemental Table S2: Major echocardiographic nomograms for junior athletes.

## References

[1] Lopez L., Colan S.D., Frommelt P.C., Ensing G.J., Kendall K., Younoszai A.K., Lai W.W., Geva T. (2010). Recommendations for quantification methods during the performance of a pediatric echocardiogram: A report from the Pediatric Measurements Writing Group of the American Society of Echocardiography Pediatric and Congenital Heart Disease Council. J. Am. Soc. Echocardiogr..

[2] Lopez L., Colan S., Stylianous M., Granger S., Trachtenberg F., Frommelt P., Pearson G., Camarda J., Cnota J., Cohen M. (2017). Relationship of Echocardiographic *Z* Scores Adjusted for Body Surface Area to Age, Sex, Race, and Ethnicity: The Pediatric Heart Network Normal Echocardiogram Database. Circ. Cardiovasc. Imaging.

[3] Cantinotti M., Scalese M., Murzi B., Assanta N., Spadoni I., Festa P., De Lucia V., Crocetti M., Marotta M., Molinaro S. (2014). Echocardiographic nomograms for ventricular, valvular and arterial dimensions in Caucasian children with a special focus on neonates, infants and toddlers. J. Am. Soc. Echocardiogr..

[4] Cantinotti M., Giordano R., Scalese M., Murzi B., Assanta N., Spadoni I., Maura C., Marco M., Molinaro S., Kutty S. (2017). Nomograms for two-dimensional echocardiography derived valvular and arterial dimensions in Caucasian children. J. Cardiol..

[5] Cantinotti M., Scalese M., Giordano R., Franchi E., Marchese P., Vicava C., Assanta N., Iervasi G., Kutty S., Koestenberger M. (2020). Pediatric nomograms for left ventricle biplane 2D volumes in healthy Caucasian children. Echocardiography.

[6] Gokhroo R.K., Anantharaj A., Bisht D., Kishor K., Plakkal N., Aghoram R., Mondal N., Pandey S.K., Roy R. (2017). A pediatric echocardiographic Z-score nomogram for a developing country: Indian pediatric echocardiography study—The Z-score. Ann. Pediatr. Cardiol..

[7] Singh V., Satheesh S., Ganapathy S., Nair N.-P.S., Mondal N., Selvaraj R., Mishra N., Anantharaj A. (2023). Echocardiographic nomograms and Z-scores for term Indian neonates. Ann. Pediatr. Cardiol..

[8] Cantinotti M., Scalese M., Contini F.V., Franchi E., Viacava C., Corana G., Pizzuto A., Pietro M., Santoro G., Assanta N. (2024). Comprehensive Two-Dimensional Pediatric Echocardiographic Nomograms for Coronary Artery Sizes in Caucasian Children and Comparison among Major Nomograms. Diagnostics.

[9] Kobayashi T., Fuse S., Sakamoto N., Mikami M., Ogawa S., Hamaoka K., Arakaki Y., Nakamura T., Nagasawa H., Kato T. (2016). A New Z Score Curve of the Coronary Arterial Internal Diameter Using the Lambda-Mu-Sigma Method in a Pediatric Population. J. Am. Soc. Echocardiogr..

[10] Dallaire F., Dahdah N. (2011). New equations and a critical appraisal of coronary artery Z scores in healthy children. J. Am. Soc. Echocardiogr..

[11] Zhang Y.Q., Chen S.B., Huang G.Y., Zhang H.Y., Huang M.R., Wang S.S., Wu L., Hong W., Shen R., Liu Y. (2015). Coronary artery indexed diameter and z score regression equations in healthy Chinese Han children. J. Clin. Ultrasound.

[12] Olivieri L., Arling B., Friberg M., Sable C. (2009). Coronary artery Z score regression equations and calculators derived from a large heterogeneous population of children undergoing echocardiography. J. Am. Soc. Echocardiogr..

[13] O’Leary P.W. (1999). Pediatric diastology: Use and limitations of Doppler echocardiography in the evaluation of ventricular diastolic function in children. Prog. Pediatr. Cardiol..

[14] Dallaire F., Slorach C., Hui W., Sarkola T., Friedberg M.K., Bradley T.J., Jaeggi E., Dragulescu A., Har R.L., Cherney D.Z. (2015). Reference values for pulse wave Doppler and tissue Doppler imaging in pediatric echocardiography. Circ. Cardiovasc. Imaging.

[15] Eidem B.W., McMahon C.J., Cohen R.R., Wu J., Finkelshteyn I., Kovalchin J.P., Ayres N.A., Bezold L.I., O’Brian Smith E., Pignatelli R.H. (2004). Impact of cardiac growth on Doppler tissue imaging velocities: A study in healthy children. J. Am. Soc. Echocardiogr..

[16] Cui W., Roberson D.A. (2006). Left ventricular Tei index in children: Comparison of tissue Doppler imaging, pulsed wave Doppler and M-mode echocardiography normal values. J. Am. Soc. Echocardiogr..

[17] Roberson D.A., Cui W., Chen Z., Madronero L.F., Cuneo B.F. (2007). Annular and septal Doppler tissue imaging in children: Normal z-score tables and effects of age, heart rate, and body surface area. J. Am. Soc. Echocardiogr..

[18] Cantinotti M., Giordano R., Scalese M., Murzi B., Assanta N., Spadoni I., Crocetti M., Marotta M., Molinaro S., Kutty S. (2016). Nomograms for mitral inflow Doppler and tissue Doppler velocities in Caucasian children. J. Cardiol..

[19] Schmitz L., Stiller B., Pees C., Koch H., Xanthopoulos A., Lange P. (2004). Doppler-derived parameters of diastolic left ventricular function in preterm infants with a birth weight <1500 g: Reference values and differences to term infants. Early Hum. Dev..

[20] Cantinotti M., Giordano R., Scalese M., Franchi E., Corana G., Assanta N., Maura C., Marco M., Molinaro S., Koestenberger M. (2018). Nomograms for echocardiographic right ventricular sub-costal view dimensions in healthy Caucasian children: A new approach to measure the right ventricle. J. Cardiol..

[21] Cantinotti M., Giordano R., Scalese M., Franchi E., Assanta N., Molinaro S., Marchese P., Paterni M., Iervasi G., Kutty S. (2019). Nomograms of pulsed Doppler velocities, times, and velocity time integrals for semilunar valves and great arteries in healthy Caucasian children. Int. J. Cardiol..

[22] Koestenberger M., Nage B., Ravekes W., Avian A., Burmas A., Grangl G., Cvirn G., Gamillscheg A. (2015). Right Ventricular Outflow Tract Velocity Time Integral Determination in 570 Healthy Children and in 52 Pediatric Atrial Septal Defect Patients. Pediatr. Cardiol..

[23] Núñez-Gil I.J., Rubio M.D., Cartón A.J., López-Romero P., Deiros L., García-Guereta L., Labrandero C., Gutiérrez-Larraya F. (2011). Determination of normalized values of the tricuspid annular plane systolic excursion (TAPSE) in 405 Spanish children and adolescents. Rev. Esp. Cardiol..

[24] Hashimoto I., Watanabe K., Kaneda H. (2015). Z-values of tricuspid annular plane systolic excursion in Japanese children. Pediatr. Int..

[25] Uysal F., Bostan Ö.M., Çil E. (2016). Determination of reference values for tricuspid annular plane systolic excursion in healthy Turkish children. Anatol. J. Cardiol..

[26] Weismann C.G., Bamdad M.C., Abraham S., Ghiroli S., Dziura J., Hellenbrand W.E. (2015). Normal pediatric data for isovolumic acceleration at the lateral tricuspid valve annulus-a heart rate—Dependent measure of right ventricular contractility. Echocardiography.

[27] Koestenberger M., Nagel B., Ravekes W., Avian A., Heinzl B., Cvirn G., Fritsch P., Fandl A., Rehak T., Gamillscheg A. (2012). Reference values of tricuspid annular peak systolic velocity in healthy pediatric patients, calculation of z score, and comparison to tricuspid annular plane systolic excursion. Am. J. Cardiol..

[28] Terada T., Mori K., Inoue M., Yasunobu H. (2016). Mitral annular plane systolic excursion/left ventricular length (MAPSE/L) as a simple index for assessing left ventricular longitudinal function in children. Echocardiography.

[29] Koestenberger M., Ravekes W., Nagel B., Avian A., Heinzl B., Cvirn G., Fritsch P., Fandl A., Rehak T., Gamillscheg A. (2014). Reference values of the right ventricular outflow tract systolic excursion in 711 healthy children and calculation of z-score values. Eur. Heart J.-Cardiovasc. Imaging.

[30] Maffessanti F., Muraru D., Esposito R., Gripari P., Ermacora D., Santoro C., Tamborini G., Galderisi M., Pepi M., Badano L.P. (2013). Age-, Body Size- and Gender-Specific Reference Values for Right Ventricular Volumes and Ejection Fraction by Three-Dimensional Echocardiography: A Multicenter Echocardiographic Study in 507 Healthy Volunteers. Circ. Cardiovasc. Imaging.

[31] Koestenberger M., Grangl G., Avian A., Gamillscheg A., Grillitsch M., Cvirn G., Burmas A., Hansmann G. (2017). Normal Reference Values and z Scores of the Pulmonary Artery Acceleration Time in Children and Its Importance for the Assessment of Pulmonary Hypertension. Circ. Cardiovasc. Imaging.

[32] Koestenberger M., Ravekes W., Everett A.D., Stueger H.P., Heinzl B., Gamillscheg A., Cvirn G., Boysen A., Fandl A., Nagel B. (2009). Right ventricular function in infants, children and adolescents: Reference values of the tricuspid annular plane systolic excursion (TAPSE) in 640 healthy patients and calculation of z score values. J. Am. Soc. Echocardiogr..

[33] Marchese P., Scalese M., Giordano R., Assanta N., Franchi E., Koestenberger M., Ravaglioli A., Kutty S., Cantinotti M. (2021). Pediatric ranges of normality for 2D speckle-tracking echocardiography atrial strain: Differences between p- and r-gating and among new (Atrial Designed) and conventional (Ventricular Specific) software’s. Echocardiography.

[34] Acheampong B., Parra D., Havens C., Jantzen D., Godown J., Soslow J. (2023). Vendor independent myocardial strain values in children. Echocardiography.

[35] Adar A., Ghelani S.J., Sleeper L.A., Lu M., Marcus E., Ferraro A.M., Colan S.D., Banka P., Powell A.J., Harrild D.M. (2019). Normal Values for Left Ventricular Strain and Synchrony in Children Based on Speckle Tracking Echocardiography. Am. J. Cardiol..

[36] Jimbo S., Noto N., Okuma H., Kato M., Komori A., Ayusawa M., Morioka I. (2020). Normal reference values for left atrial strains and strain rates in school children assessed using two-dimensional speckle-tracking echocardiography. Heart Vessels.

[37] Harrington J.K., Ferraro A.M., Colan S.D., Sleeper L.A., Lu M., Adar A., Powell A.J., Levy P.T., Harrild D.M. (2021). Normal Left Ventricular Systolic and Diastolic Strain Rate Values in Children Derived from Two-Dimensional Speckle-Tracking Echocardiography. J. Am. Soc. Echocardiogr..

[38] Kotby A.A., Ebrahim S.O.S., Al-Fahham M.M. (2023). Reference centiles for left ventricular longitudinal global and regional systolic strain by automated functional imaging in healthy Egyptian children. Cardiol. Young.

[39] Davarpasand T., Jalali A., Mohseni-Badalabadi R., Toofaninejad N., Hali R., Fallah F., Seilani P., Hosseinsabet A. (2024). Normal ranges of left atrial phasic strains and strain rates by 2D speckle-tracking echocardiography in pediatrics: A systematic review and meta-analysis. Sci. Rep..

[40] Koopman L.P., Rebel B., Gnanam D., Menting M.E., Helbing W.A., Boersma E. (2019). Reference values for two-dimensional myocardial strain echocardiography of the left ventricle in healthy children. Cardiol. Young.

[41] Zhang L., Gao J., Xie M., Yin P., Liu W., Li Y., Klas B., Sun J., Balluz R., Ge S. (2013). Left ventricular three-dimensional global systolic strain by real-time three-dimensional speckle-tracking in children: Feasibility, reproducibility, maturational changes, and normal ranges. J. Am. Soc. Echocardiogr..

[42] Kamel H., Elsayegh A.T., Nazmi H., Attia H.M. (2022). Assessment of left ventricular systolic function using two- and three-dimensional speckle tracking echocardiography among healthy preschool-age pediatric children. Egypt. Heart J..

[43] Aristizábal-Duque C.H., Fernández Cabeza J., Blancas Sánchez I.M., Delgado Ortega M., Aparicio Martinez P., Romero-Saldaña M., del Pozo F.J.F., Pan M., Ruiz Ortiz M., Mesa-Rubio M.D. (2022). The Assessment of Myocardial Longitudinal Strain in a Paediatric Spanish Population Using a New Software Analysis. J. Clin. Med..

[44] Marcus K.A., Mavinkurve-Groothuis A.M., Barends M., van Dijk A., Feuth T., de Korte C., Kapusta L. (2011). Reference values for myocardial two-dimensional strain echocardiography in a healthy pediatric and young adult cohort. J. Am. Soc. Echocardiogr..

[45] Klitsie L.M., Roest A.A., van der Hulst A.E., Stijnen T., Blom N.A., Ten Harkel A.D. (2013). Assessment of intraventricular time differences in healthy children using two-dimensional speckle tracking echocardiography. J. Am. Soc. Echocardiogr..

[46] Dallaire F., Slorach C., Bradley T., Hui W., Sarkola T., Friedberg M.K., Jaeggi E., Dragulescu A., Mahmud F.H., Daneman D. (2016). Pediatric Reference Values and Z Score Equations for Left Ventricular Systolic Strain Measured by Two-Dimensional Speckle-Tracking Echocardiography. J. Am. Soc. Echocardiogr..

[47] Cantinotti M., Scalese M., Giordano R., Franchi E., Assanta N., Marotta M., Viacava C., Molinaro S., Iervasi G., Santoro G. (2018). Normative Data for Left and Right Ventricular Systolic Strain in Healthy Caucasian Italian Children by Two-Dimensional Speckle-Tracking Echocardiography. J. Am. Soc. Echocardiogr..

[48] Levy P.T., Mejia A.A.S., Machefsky A., Fowler S., Holland M.R., Singh G.K. (2014). Normal ranges of right ventricular systolic and diastolic strain measures in children: A systematic review and meta-analysis. J. Am. Soc. Echocardiogr..

[49] Ghelani S.J., Brown D.W., Kuebler J.D., Perrin D., Shakti D., Williams D.N., Marx G.R., Colan S.D., Geva T., Harrild D.M. (2018). Left atrial volumes and strain in healthy children measured by three-dimensional echocardiography: Normal values and maturational changes. J. Am. Soc. Echocardiogr..

[50] Kutty S., Padiyath A., Li L., Peng Q., Rangamani S., Schuster A., Danford D.A. (2013). Functional Maturation of left and right atrial systolic and diastolic performance in infants, children, and adolescents. J. Am. Soc. Echocardiogr..

[51] Ramlogan S., Aly D., France R., Schmidt S., Hinzman J., Sherman A., Goudar S.P., Forsha D. (2020). Reproducibility and Intervendor Agreement of Left Ventricular Global Systolic Strain in Children Using a Layer-Specific Analysis. J. Am. Soc. Echocardiogr..

[52] Romanowicz J., Ferraro A.M., Harrington J.K., Sleeper L.A., Adar A., Levy P.T., Powell A.J., Harrild D.M. (2023). Pediatric Normal Values and Z Score Equations for Left and Right Ventricular Strain by Two-Dimensional Speckle-Tracking Echocardiography Derived from a Large Cohort of Healthy Children. J. Am. Soc. Echocardiogr..

[53] Amedro P., Bredy C., Guillaumont S., De La Villeon G., Gamon L., Lavastre K., Meli A.C., Richard S., Cazorla O., Lacampagne A. (2019). Speckle tracking echocardiography in healthy children: Comparison between the QLAB by Philips and the EchoPAC by General Electric. Int. J. Cardiovasc. Imaging.

[54] Ferraro A.M., Adar A., Ghelani S.J., Sleeper L.A., Levy P.T., Rathod R.H., Marx G.R., Harrild D.M. (2020). Speckle tracking echocardiographically-based analysis of ventricular strain in children: An intervendor comparison. Cardiovasc. Ultrasound.

[55] Palmer C., Truong V.T., Klas B., Wolking S., Ornella A., Young M., Ngo T.N.M., Tretter J.T., Nagueh S.F., Mazur W. (2020). Left and right atrial speckle tracking: Comparison of three methods of time reference gating. Echocardiography.

[56] Badano L.P., Kolias T.J., Muraru D., Abraham T.P., Aurigemma G., Edvardsen T., D’Hooge J., Donal E., Fraser A.G., Marwick T. (2018). Standardization of left atrial, right ventricular, and right atrial deformation imaging using two-dimensional speckle tracking echocardiography: A consensus document of the EACVI/ASE/Industry Task Force to standardize deformation imaging. Eur. Heart J. Cardiovasc. Imaging.

[57] Voigt J.U., Pedrizzetti G., Lysyansky P., Marwick T.H., Houle H., Baumann R., Pedri S., Ito Y., Abe Y., Metz S. (2015). Definitions for a common standard for 2D speckle tracking echocardiography: Consensus document of the EACVI/ASE/Industry Task Force to standardize deformation imaging. J. Am. Soc. Echocardiogr..

[58] Kuebler J.D., Ghelani S., Williams D.M., Nathan M., Marx G., Colan S.D., Harrild D.M. (2018). Normal Values and Growth-Related Changes of Left Ventricular Volumes, Stress, and Strain in Healthy Children Measured by 3-Dimensional Echocardiography. Am. J. Cardiol..

[59] Jone P.N., Le L., Pan Z., Colen T., Shigemitsu S., Khoo N.S., Goot B.H., Parthiban A., Harrild D.M., Ferraro A.M. (2021). A multicenter study of three-dimensional echocardiographic evaluation of normal pediatric left ventricular volumes and function. Echocardiography.

[60] Krell K., Laser K.T., Dalla-Pozza R., Winkler C., Hildebrandt U., Kececioglu D., Breuer J., Herberg U. (2018). Real-time three-dimensional echocardiography of the left ventricle—Pediatric percentiles and head-to-head comparison of different contour finding algorithms: A multicenter study. J. Am. Soc. Echocardiogr..

[61] Herberg U., Smit F., Winkler C., Dalla-Pozza R., Breuer J., Laser K.T. (2021). Real-time 3D-echocardiography of the right ventricle—Paediatric reference values for right ventricular volumes using knowledge-based reconstruction: A multicentre study. Quant. Imaging Med. Surg..

[62] Cantinotti M., Scalese M., Giordano R., Franchi E., Marchese P., Assanta N., Molinaro S., Paterni M., Iervasi G., Koestenberger M. (2019). Three-Dimensional Echocardiography Derived Nomograms for Left Ventricular Volumes in Healthy Caucasian Italian Children. J. Am. Soc. Echocardiogr..

[63] Jone P.N., Schäfer M., Pan Z., Bremen C., Ivy D.D. (2018). 3D echocardiographic evaluation of right ventricular function and strain: A prognostic study in paediatric pulmonary hypertension. Eur. Heart J. Cardiovasc. Imaging.

[64] Linden K., Goldschmidt F., Laser K.T., Winkler C., Körperich H., Dalla-Pozza R., Breuer J., Herberg U. (2019). Left Atrial Volumes and Phasic Function in Healthy Children: Reference Values Using Real-Time Three-Dimensional Echocardiography. J. Am. Soc. Echocardiogr..

[65] Sabatino J., Borrelli N., Fraisse A., Herberg J., Karagadova E., Avesani M., Bucciarelli V., Josen M., Paredes J., Piccinelli E. (2021). Abnormal myocardial work in children with Kawasaki disease. Sci. Rep..

[66] Sabatino J., Leo I., Strangio A., La Bella S., Borrelli N., Avesani M., Josen M., Paredes J., Piccinelli E., Sirico D. (2022). Echocardiographic Normal Reference Ranges for Non-invasive Myocardial Work Parameters in Pediatric Age: Results from an International Multi-Center Study. Front. Cardiovasc. Med..

[67] Cui C., Zheng Q., Li Y., Huang D., Hu Y., Wang Y., Liu R., Liu L., Zhang L. (2022). Reference Values of Noninvasive Myocardial Work Indices Measured by Echocardiography in Healthy Children. Front. Pediatr..

[68] Pham T.T.M., Truong V.T., Vu P.N., Tran T.X., Nguyen N.N.H., Nguyen L.P.T., Tu H.N.T., Palmer C., Tretter J.T., Levy P. (2022). Echocardiographic Reference Ranges of Non-invasive Myocardial Work Indices in Children. Pediatr. Cardiol..

[69] Luo X., Ge Q., Su J., Zhou N., Li P., Xiao X., Chen Y., Wang D., Ma Y., Ma L. (2022). Normal ranges of non-invasive left ventricular myocardial work indices in healthy young people. Front. Pediatr..

[70] Tretter J.T., Pradhan S., Truong V.T., Mullikin A., Mazur W., Hill G.D., Redington A.N., Taylor M.D. (2021). Non-invasive left ventricular myocardial work indices in healthy adolescents at rest. Int. J. Cardiovasc. Imaging.

[71] Marchese P., Scalese M., Assanta N., Franchi E., Viacava C., Santoro G., Corana G., Pizzuto A., Contini F.V., Kutty S. (2024). Normal Values for Echocardiographic Myocardial Work in a Large Pediatric Population. Diagnostics.

[72] Cantinotti M., Scalese M., Giordano R., Assanta N., Marchese P., Franchi E., Viacava C., Koestenberger M., Jani V., Kutty S. (2021). A statistical comparison of reproducibility in current pediatric two-dimensional echocardiographic nomograms. Pediatr. Res..

[73] https://zscore.chboston.org/.

[74] https://parameterz.com.

[75] https://www.pediatricheartnetwork.org/z-scores-calculator/.

[76] https://www.prisma-statement.org/.

[77] Lopez L., Saurers D.L., Barker P.C.A., Cohen M.S., Colan S.D., Dwyer J., Forsha D., Friedberg M.K., Lai W.W., Printz B.F. (2024). Guidelines for Performing a Comprehensive Pediatric Transthoracic Echocardiogram: Recommendations from the American Society of Echocardiography. J. Am. Soc. Echocardiogr..

[78] Simpson J., Lopez L., Acar P., Friedberg M.K., Khoo N.S., Ko H.H., Marek J., Marx G., McGhie J.S., Meijboom F. (2017). Three-dimensional Echocardiography in Congenital Heart Disease: An Expert Consensus Document from the European Association of Cardiovascular Imaging and the American Society of Echocardiography. J. Am. Soc. Echocardiogr..

[79] Mahgerefteh J., Lai W., Colan S., Trachtenberg F., Gongwer R., Stylianou M., Bhat A.H., Goldberg D., McCrindle B., Frommelt P. (2021). Height Versus Body Surface Area to Normalize Cardiovascular Measurements in Children Using the Pediatric Heart Network Echocardiographic Z-Score Database. Pediatr. Cardiol..

[80] Williams K., Thomson D., Seto I., Contopoulos-Ioannidis D.G., Ioannidis J.P., Curtis S., Constantin E., Batmanabane G., Hartling L., Klassen T. (2012). Standard 6: Age groups for pediatric trials. Pediatrics.

[81] Thompson W., Endriss J. (1961). The required sample size when estimating variances. Am. Stat..

[82] Kish L. (1965). Survey Sampling.

[83] Plante V., Gobeil L., Xiong W.T., Touré M., Dahdah N., Greenway S.C., Drolet C., Wong K.K., Mackie A.S., Bradley T.J. (2021). Alternative to Body Surface Area as a Solution to Correct Systematic Bias in Pediatric Echocardiography Z Scores. Can. J. Cardiol..

[84] Haycock G.B., Schwartz G.J., Wisotsky D.H. (1978). Geometric method for measuring body surface area: A height-weight formula validated in infants, children, and adults. J. Pediatr..

[85] Bonatto R.C., Fioretto J.R., Okoshi K., Matsubara B.B., Padovani C.R., Manfrin T.C.R., Gobbi M., Martino R.S., Bregagnollo E.A. (2006). Percentile curves of normal values of echocardiographic measurements in normal children from the central-southern region of the State of Sao Paulo, Brazil. Arq. Bras. Cardiol..

[86] White H.A. (1980). Heteroscedasticity-consistent covariance matrix estimator and a direct test for heteroscedasticity. Econometrica.

[87] Breusch T.S., Pagan A.R. (1979). A Simple test for heteroscedasticity and random coefficient variation. Econometrica.

[88] Shapiro S.S., Wilk M.B. (1965). An Analysis of Variance Test for Normality (Complete Samples). Biometrika.

[89] Lilliefors H. (1967). On the Kolmogorov–Smirnov test for normality with mean and variance unknown. J. Am. Stat. Assoc..

[90] Sluysmans T., Colan S.D. (2005). Theoretical and empirical derivation of cardiovascular allometric relationships in children. J. Appl. Physiol..

[91] Pettersen M.D., Du W., Skeens M.E., Humes R.A. (2008). Regression equations for calculation of z scores of cardiac structures in a large cohort of healthy infants, children, and adolescents: An echocardiographic study. J. Am. Soc. Echocardiogr..

[92] Majonga E.D., Rehman A.M., McHugh G., Mujuru H.A., Nathoo K., Patel M.S., Munyati S., Odland J.O., Kranzer K., Kaski J.P. (2017). Echocardiographic reference ranges in older children and adolescents in sub-Saharan Africa. Int. J. Cardiol..

[93] Majonga E.D., Norrish G., Rehman A.M., Kranzer K., Mujuru H.A., Nathoo K., Odland J.O., Kaski J.P., Ferrand R.A. (2018). Racial Variation in Echocardiographic Reference Ranges for Left Chamber Dimensions in Children and Adolescents: A Systematic Review. Pediatr. Cardiol..

[94] Zilberman M.V., Khoury P.R., Kimball R.T. (2005). Two-dimensional echocardiographic valve measurements in healthy children: Gender-specific differences. Pediatr. Cardiol..

[95] Gautier M., Detaint D., Fermanian C., Aegerter P., Delorme G., Arnoult F., Milleron O., Raoux F., Stheneur C., Boileau C. (2010). Nomograms for aortic root diameters in children using two-dimensional echocardiography. Am. J. Cardiol..

[96] Cantinotti M., Scalese M., Murzi B., Assanta N., Spadoni I., De Lucia V., Crocetti M., Cresti A., Gallotta M., Marotta M. (2014). Echocardiographic nomograms for chamber diameters and areas in Caucasian children. J. Am. Soc. Echocardiogr..

[97] Ciccone M.M., Scicchitano P., Zito A., Gesualdo M., Sassara M., Calderoni G., Di Mauro F., Ladisa G., Di Mauro A., Laforgia N. (2011). Different functional cardiac characteristics observed in term/preterm neonates by echocardiography and tissue Doppler imaging. Early Hum. Dev..

[98] Lorch S.M., Ludomirsky A., Singh G.K. (2008). Maturational and growth-related changes in left ventricular longitudinal strain and strain rate measured by two-dimensional speckle tracking echocardiography in healthy pediatric population. J. Am. Soc. Echocardiogr..

[99] Cantinotti M., Kutty S., Giordano R., Assanta N., Murzi B., Crocetti M., Marotta M., Iervasi G. (2015). Review and status report of pediatric left ventricular systolic strain and strain rate nomograms. Heart Fail. Rev..

[100] Takahashi K., Al Naami G., Thompson R., Inage A., Mackie A.S., Smallhorn J.F. (2010). Normal rotational, torsion and untwisting data in children, adolescents and young adults. J. Am. Soc. Echocardiogr..

[101] Kaku K., Takeuchi M., Tsang W., Takigiku K., Yasukochi S., Patel A.R., Mor-Avi V., Lang R.M., Otsuji Y. (2014). Age-related normal range of left ventricular strain and torsion using three-dimensional speckle-tracking echocardiography. J. Am. Soc. Echocardiogr..

[102] Kim H.J., Yoon J.H., Lee E.J., Oh J.H., Lee J.Y., Lee S.J., Han J.W. (2015). Normal left ventricular torsion mechanics in healthy children: Age related changes of torsion parameters are closely related to changes in heart rate. Korean Circ. J..

[103] Poutanen T., Jokinen E. (2007). Left ventricular mass in 169 healthy children and young adults assessed by three-dimensional echocardiography. Pediatr. Cardiol..

[104] Boettler P., Hartmann M., Watzl K., Maroula E., Schulte-Moenting J., Knirsch W., Dittrich S., Kececioglu D. (2005). Heart rate effects on strain and strain rate in healthy children. J. Am. Soc. Echocardiogr..

[105] Poutanen T., Jokinen E., Sairanen H., Tikanoja T. (2003). Left atrial and left ventricular function in healthy children and young adults assessed by three dimensional echocardiography. Heart.

[106] Poutanen T., Tikanoja T., Sairanen H., Jokinen E. (2006). Normal mitral and aortic valve areas assessed by three- and two-dimensional echocardiography in 168 children and young adults. Pediatr. Cardiol..

[107] Lu D.F., Tong X.M., Liu Y.F., Zhang H. (2022). Reference Values for Point-of-Care Echocardiographic Measurements of Preterm Infants in China. Front. Pediatr..

[108] Ashrafi A.H., Lai W., Gaffar S., Renella P. (2021). Normative Echocardiographic Values for Right and Left Ventricular Function in Extremely Premature Neonates. J. Pediatr..

[109] Wang S., Fu J., Wu L., Liu X.Y., Zhang Y. (2022). Percentile curves of normal echocardiographic measurements values for left heart structures in 1570 Han Chinese preterm and term infants. J. Clin. Ultrasound.

[110] Calado C., Collins C., Drew S., Holberton J. (2019). Reference echocardiographic measurements in very low birth weight preterm infants. Am. J. Perinatol..

[111] Skelton R., Gill A.B., Parsons J.M. (1998). Reference ranges for cardiac dimensions and blood flow velocity in preterm infants. Heart.

[112] Abushaban L., Vel M.T., Rathinasamy J., Sharma P.N. (2017). Normal reference ranges for pulmonary artery diameters in preterm infants. Pediatr. Cardiol..

[113] Choudhry S., Salter A., Cunningham T.W., Levy P.T., Nguyen H.H., Wallendorf M., Singh G.K., Johnson M.C. (2017). Normative Left Ventricular M-Mode Echocardiographic Values in Preterm Infants up to 2 kg. J. Am. Soc. Echocardiogr..

[114] Krysztofiak H., Młyńczak M., Folga A., Braksator W., Małek Ł.A. (2019). Normal values for left ventricular mass in relation to lean body mass in child and adolescent athletes. Pediatr. Cardiol..

[115] Cavarretta E., Maffessanti F., Sperandii F., Guerra E., Quaranta F., Nigro A., Minati M., Rebecchi M., Fossati C., Calò L. (2018). Reference values of left heart echocardiographic dimensions and mass in male peri-pubertal athletes. Eur. J. Prev. Cardiol..

[116] Sharma S., Maron B.J., Whyte G., Firoozi S., Elliott P.M., McKenna W.J. (2002). Physiologic limits of left ventricular hypertrophy in elite junior athletes: Relevance to differential diagnosis of athlete’s heart and hypertrophic cardiomyopathy. J. Am. Coll. Cardiol..

[117] Makan J., Sharma S., Firoozi S., Whyte G., Jackson P.G., McKenna W.J. (2005). Physiological upper limits of ventricular cavity size in highly trained adolescent athletes. Heart.

[118] George K., Sharma S., Batterham A., Whyte G., McKenna W. (2001). Allometric analysis of the association between cardiac dimensions and body size variables in 464 junior athletes. Clin. Sci..

[119] Chen L., Chen W., Zhu Y., Zhang Z., Liu T., Zhang L. (2025). Global research landscape on artificial intelligence in echocardiography from 1997 to 2024: Bibliometric analysis. Digit. Health.

[120] Mayourian J., Asztalos I.B., El-Bokl A., Lukyanenko P., Kobayashi R.L., La Cava W.G., Ghelani S.J., Vetter V.L., Triedman J.K. (2025). Electrocardiogram-based deep learning to predict left ventricular systolic dysfunction in paediatric and adult congenital heart disease in the USA: A multicentre modelling study. Lancet Digit. Health.

