# Supplementary material for: Pediatric Echocardiographic Nomograms: Twenty Years of Advances—Do We Now Have a Complete and Reliable Tool, or Are Gaps Still Present? An Up-to-Date Review
Source: J Clin Med. 2025 Jul 23;14(15):5215. doi: 10.3390/jcm14155215 (PMC12347125; doi:10.3390/jcm14155215)
Supplement: Supplementary file 1 [file jcm-14-05215-s001.zip › jcm-3655801-supplementary.pdf]

Supplemental tables

Supplemental Table S1: major pediatric nomograms for functional assessment of the right ventricle by echocardiography.

**Author**	**Sample size**	**Parameters**	**Data normalization**	**Data expression**
Núñez-Gil<br>Spain 2015 (23)	n. 405<br>0-18 yrs	TAPSE	Age Groups	Mean ± SD
Hashimoto<br>Japan 2015 (24)	n. 953<br>0-22.7 yrs	TAPSE	Age groups and BSA (formula not specified)	Z score
Uysal F<br>Turkey 2016 (25)	n. 756<br>0-18 yrs	TAPSE	Age groups and BSA Mosteller	Z score
Weismann (26)<br>USA 2016	n. 340<br>0-18 yrs	TV S', TAPSE	Heart Rate	Z score
Köstenberger<br>Austria 2009 (32)	n. 640<br>0-18 yrs	TASPE	BSA Mosteller	Z score
Köstenberger<br>Austria 2012 (27)	n. 860<br>0-18 yrs	TV S'	BSA Mosteller	Z score
Köstenberger<br>Austria 2013 (29)	n. 711<br>0-18 yrs	RVOT SE	BSA Mosteller	Z score
Köstenberger<br>Austria 2014 (22)	n.570<br>0-18 yrs	RVOT VTI	BSA Mosteller	Z score
Köstenberger M Austria 2017 (31)	n.756<br>0-18 yrs	PAAT	BSA Mosteller	Z score

PAAT=pulmonary artery acceleration time, RVOT= right ventricle outflow tract systolic excursion, TAPSE= Tricuspid annular plane systolic excursion, TV S' = tricuspid valve septal valve annulus TDI s' velocity, VTI=velocity time integral,

Supplemental Table S2: Major echocardiographic nomograms for junior athlete

**Author**	**Sample Size**	**Parameters**	**Normalized for**	**Data expression**
Krysztofiak et al.,<br>2018, Poland<br>(114)	n. 791 (664 M)	M-mode: LVM	Lean body mass	Z scores
Cavarretta et al.,<br>2018, Italy<br>(115)	n. 2151 (All M)<br>8-18 yrs	M-mode: LVIDd, LVIDs, LVM, Ao root, LAD, IVSd, LVPWd, FS	Age, BSA	Z scores<br>Mean ± SD
George et al.,<br>2001, UK (118)	n. 464 (335 M)<br>14- 18 yrs	M-mode: LVIDd, LVM, LAD, IVSd, LVPWd, RVIDd	Age, gender,	Mean ± SD
Makan et al.,<br>2004, UK (117)	n. 900 (664 M)<br>14- 18 yrs	Short axis: LVIDd	Age, gender	Mean ± SD<br>Upper limit
Sharma et al.,<br>2002, UK and USA (116)	n. 750 elite (540 M)<br>14- 18 yrs	Short axis: LVWT maximal wall thickness at end-diastole	Age, gender	Mean ± SD<br>Upper limit

Ao=aortic, BSA=body surface area, Fs=fraction of shortening, IVSd= interventricular septum diameter, LAD=left atrium dimension, LVIDs=left ventricle internal diameter dimension, LVWT=left ventricular wall thickness, LVPWd= left ventricle posterior wall diameter, LVM=left ventricular mass, RVIDd=right ventricle internal diameter diastolic, SD=standard deviation
